# *N*6-Methyladenosine RNA modification in cerebrospinal fluid as a novel potential diagnostic biomarker for progressive multiple sclerosis

**DOI:** 10.1186/s12967-021-02981-5

**Published:** 2021-07-22

**Authors:** Fei Ye, Tianzhu Wang, Xiaoxin Wu, Jie Liang, Jiaoxing Li, Wenli Sheng

**Affiliations:** 1 Department of Neurology, The First Affiliated Hospital, Sun Yat-Sen University, Guangzhou, China; 2 Guangdong Provincial Key Laboratory of Diagnosis and Treatment of Major Neurological Diseases, The First Affiliated Hospital, Sun Yat-Sen University, Guangzhou, China; 3 Department of Neurology, The First Affiliated Hospital of Chongqing Medical University, Chongqing, China

**Keywords:** Progressive multiple sclerosis (PMS), *N*6-methyladenosine (m6A), Cerebrospinal fluid (CSF), Diagnostic biomarker

## Abstract

**Background:**

Progressive multiple sclerosis (PMS) is an uncommon and severe subtype of MS that worsens gradually and leads to irreversible disabilities in young adults. Currently, there are no applicable or reliable biomarkers to distinguish PMS from relapsing–remitting multiple sclerosis (RRMS). Previous studies have demonstrated that dysfunction of *N*6-methyladenosine (m6A) RNA modification is relevant to many neurological disorders. Thus, the aim of this study was to explore the diagnostic biomarkers for PMS based on m6A regulatory genes in the cerebrospinal fluid (CSF).

**Methods:**

Gene expression matrices were downloaded from the ArrayExpress database. Then, we identified differentially expressed m6A regulatory genes between MS and non-MS patients. MS clusters were identified by consensus clustering analysis. Next, we analyzed the correlation between clusters and clinical characteristics. The random forest (RF) algorithm was applied to select key m6A-related genes. The support vector machine (SVM) was then used to construct a diagnostic gene signature. Receiver operating characteristic (ROC) curves were plotted to evaluate the accuracy of the diagnostic model. In addition, CSF samples from MS and non-MS patients were collected and used for external validation, as evaluated by an m6A RNA Methylation Quantification Kit and by real-time quantitative polymerase chain reaction.

**Results:**

The 13 central m6A RNA methylation regulators were all upregulated in MS patients when compared with non-MS patients. Consensus clustering analysis identified two clusters, both of which were significantly associated with MS subtypes. Next, we divided 61 MS patients into a training set (n = 41) and a test set (n = 20). The RF algorithm identified eight feature genes, and the SVM method was successfully applied to construct a diagnostic model. ROC curves revealed good performance. Finally, the analysis of 11 CSF samples demonstrated that RRMS samples exhibited significantly higher levels of m6A RNA methylation and higher gene expression levels of m6A-related genes than PMS samples.

**Conclusions:**

The dynamic modification of m6A RNA methylation is involved in the progression of MS and could potentially represent a novel CSF biomarker for diagnosing MS and distinguishing PMS from RRMS in the early stages of the disease.

**Supplementary Information:**

The online version contains supplementary material available at 10.1186/s12967-021-02981-5.

## Background

Multiple sclerosis (MS) is a complex and disabling disease of the central nervous system (CNS). Disease onset typically occurs between the ages of 20 and 50 years and is driven by complex interactions between underlying genetic and environmental factors [1]. Approximately 80–85% of patients with MS experience a natural course of relapse and remission at disease onset that is referred to as relapsing–remitting MS (RRMS) [1]. Most cases progress steadily into a secondary-progressive disease course after decades without superimposed remissions, a condition known as secondary-progressive MS (SPMS) [2]. Approximately 10–15% of patients initially present with a gradually increasing and irreversible deterioration of neurological functions; this condition is referred to as primary-progressive MS (PPMS) [3]. Although recent studies have shown that low vitamin D concentration, cigarette smoking, and obesity are highly associated with MS, the exact etiology and pathogenesis of MS has yet to be elucidated [1]. The current diagnostic criteria are beneficial to identify MS patients by integrated analysis of clinical manifestations, imaging characteristics, and cerebrospinal fluid (CSF) abnormalities [4]. However, compared to RRMS, the diagnosis of progressive MS (PMS) is usually delayed because of a retrospective history; furthermore, the available disease modifying drugs (DMDs) fail to provide benefit, eventually leading to a poor prognosis [5]. Therefore, there is a need to discover a novel biomarker for the early and accurate diagnosis of PMS to enhance survival with personalized therapeutic management.

*N*6-Methyladenosine (m6A) is the most common RNA methylation modification and is defined as methylation of the nitrogen-6 position of adenosine in the mRNA via various m6A modification regulators [6]. The specific methylation of mRNA not only influences molecular structure and mRNA-protein interactions, it also causes changes in RNA metabolism and functions. Recent studies have confirmed that neurodegeneration is accelerated in PMS lesions [7], and that dysfunctional RNA modification is related to disease course and reflects prognosis in neurodegenerative diseases such as Alzheimer’s disease (AD) and Parkinson’s disease (PD) [8, 9]. However, to our knowledge, the role of dysfunctional RNA modification in MS has not yet been reported. Recent research has identified novel biomarkers for these diseases that are usually detected in the CSF, including beta-amyloid 42, alpha-synuclein, and oligoclonal bands (OB). Therefore, the aim of the present study was to investigate diagnostic CSF biomarkers for PMS patients based on m6A regulatory genes.

## Methods

### Data download and preprocessing

First, we screened the Gene Expression Omnibus (GEO) database (https://www.ncbi.nlm.nih.gov/geo/) and the ArrayExpress database (https://www.ebi.ac.uk/arrayexpress/) for gene array expression files relating to MS using “*Homo sapiens*” (organism), “CSF” (sample), and “RNA assay” (experimental type) as the search criteria. We successfully identified two gene expression microarray datasets: E-MTAB-69 [10] and E-MTAB-2374 [11]) from the ArrayExpress database. No datasets were downloaded from the GEO database. The E-MTAB-69 dataset included the molecular profiles of 26 samples from MS cases and 18 samples from non-MS controls (non-inflammatory neurological disorders) while the E-MTAB-2374 dataset contained the expression profiles of 35 MS cases and 13 non-MS controls (non-inflammatory neurological disease controls, including stroke, neurosarcoidosis, and PD). Experiments were conducted on the Affymetrix GeneChip Human Genome U133 Plus 2.0 (*GPL570 Platform, Affymetrix, Inc*). The corresponding annotation file was used to convert identification probes into gene symbols. Mean values were used to determine the gene expression levels when several probes targeted a single gene. The robust multi-array average (RMA) algorithm was then used to obtain log2 converted and standardized mRNA expression data. Given the limited number of CSF samples from patients with MS, we performed batch-normalization to merge both gene expression profiles; the inter-batch difference was then removed by the *sva* package [12]. Subsequently, a density plot was plotted to evaluate the effectiveness of removing the inter-batch difference.

### Selection of m6A RNA methylation regulators and differential expression analysis

A total of 13 currently recognized m6A RNA methylation regulators were extracted for subsequent analysis; these included erasers (ALKBH5 and FTO); readers (HNRNPC, YTHDC1, YTHDC2, YTHDF1, and YTHDF2); and writers (KIAA1429, METTL3, METTL14, RBM15, WTAP, and ZC3H13) [13]. Next, we screened these m6A-related genes to identify differentially expressed genes (DEGs) between MS patients and non-MS controls using the empirical Bayes (eBayes) methods and the *limma* package. In this study, the cut-offs were set at a log_2_|fold change (FC)| > 1 and a false discovery rate (FDR) < 0.05 to select the DEGs between the MS and non-MS patients. In addition, the Mann–Whitney* U* test (Wilcoxon rank-sum test) was used to confirm significant m6A-related genes between MS and non-MS patients. We also constructed a box-plot of DEGs encoding m6A RNA methylation regulators. Spearman correlation analysis was then carried out to demonstrate interactive associations between each of the m6A-related genes.

### Gene functional enrichment analyses

Gene Oncology (GO) annotations were then used to determine the biological processes, cellular components, and molecular functions of the identified DEGs and differentially expressed m6A-related genes [14]. The integrated molecular pathways of these genes were also acquired from the Kyoto Encyclopedia of Genes and Genomes (KEGG) database [15]. The significant GO terms and KEGG pathways were considered enrichments when using a specific cut-off (FDR < 0.05) via the *org.Hs.eg.db*, *clusterProfiler*, and *GOplot* packages. In addition, protein–protein interaction (PPI) networks were constructed with a high confidence (> 0.7) using the Search Tool for the Retrieval of Interacting Genes (STRING) database [16].

### Consensus clustering analysis

Next, we investigated the link between m6A RNA methylation regulators and MS classification by clustering the merged dataset into different subgroups using the *ConsensusClusterPlus* package. The unsupervised clustering method was applied to identify different clusters. The clustering algorithm was partitioned around medoids and distances were measured by the Euclidean metric system. Principal component analysis (PCA) was then conducted to verify the classification results. Consequently, the difference in both m6A-related genes and clinical parameters between these clusters were determined using the Chi-square test; these were subsequently presented as a heatmap.

### Generation of a random forest (RF) algorithm for feature gene selection and a support vector machine (SVM) classifier

The integrated dataset was randomly divided into a training set for model development (2/3, n = 41) and a test set for model validation (1/3, n = 20) using the *caret* package. For feature gene selection, the RF algorithm was used to rank the importance of these m6A-related genes; to do this, we used the *randomForest* package. The m6A-related feature genes were identified based on a relative importance > 0.4. During model development, we used the selected feature genes to establish a SVM classifier with a C-type classification and a radial basis function kernel. This was measured with a fivefold cross validation via the *e1071* package. Patients were then classified and differentiated by gene expression levels. We also used eigen values to predict the probabilities of patients belonging to the same classification; these values could distinguish and predict the different subtypes of MS. During model validation, we used the test set to verify our previous findings. The area under the receiver operating characteristic (ROC) curve was used to evaluate the effect of classification for both the training and test sets. Moreover, a correlation-based SVM filter was also applied to confirm the feature genes. PCA analysis was applied to evaluate the performance of the correlation-based distances for clustering.

### External validation of m6A RNA methylation in MS patients

This study was approved by the Independent Ethics Committee of the First Affiliated Hospital of Sun Yat-sen University, and all patients signed an informed consent form. Patients with a clinical diagnosis of MS were enrolled for external validation between July 2020 and December 2020 at the First Affiliated Hospital, Sun Yat-sen University. The diagnostic criteria were based on the 2017 revisions of the McDonald criteria [4]. Patients who were diagnosed with other autoimmune diseases and had malignant tumors were excluded from this study. Consequently, these patients were automatically divided into RRMS and PMS subgroups. In addition, patients diagnosed with other neurodegenerative disorders between May 2021 and June 2021 were also recruited to act as non-MS controls. A 2 mL sample of CSF was collected via lumbar puncture and placed in a 5-mL RNase/DNase-free centrifuge tube. We excluded CSF samples if they were contaminated by blood. Samples were immediately centrifuged at 500×*g* for 10 min at 4 °C (ThermoFisher, Sorvall ST40R, USA). Next, total RNA was extracted and purified from CSF cells using the RNeasy Micro Kit (GIAGEN, 74004, Germany) in accordance with the manufacturer’s protocol. In brief, CSF cells were disrupted with 350 µl of Buffer RLT, mixed with 350 µl of 70% ethanol, and transferred to a 2 ml RNeasy MinElute spin column, retrospectively. The spin column membrane was then washed with 350 µl of Buffer RW1, and DNA was eliminated by incubation with DNase I mix (10 µl of DNase I stock solution and 70 µl of Buffer RDD) at room temperature (RT) for 15 min. The RNA was then purified with 500 µl of Buffer RPE and 500 µl of 80% ethanol. The flowthrough was removed by centrifugation at 8000×*g* for 15 s. Next, 14 µl of RNase-free water was added to the center of a spin column membrane in order to isolate the RNA. The ratio of absorbance at 260 and 280 nm (A260/A280) was calculated to evaluate the purity of RNA, with a cut-off value of 2.0 (ThermoFisher, NanoDrop One, USA). The extracted RNA was then stored at − 80 °C to await further analysis.

First, we measured the global m6A levels in total CSF cell RNA with an m6A RNA Methylation Quantification Kit (Fluorometric; Abcam, ab233491, UK) in accordance with the manufacturer’s protocol. Because of the very low and highly variable RNA yield from CSF samples, we used 100 ng of total RNA from each sample. In brief, 2 µl of negative control, 2 µl of diluted positive control, and 100 ng of RNA samples, were added to each well and incubated at 37 °C for 90 min after adding 80 µl of binding solution to the 96-well plate. Then, m6A RNA was captured by covering and incubating 50 µl of diluted Capture Antibody at RT for 60 min, 50 µl of diluted Detection Antibody for 30 min, and 50 µl of diluted Enhancer Solution for 30 min, retrospectively. Each well was washed three times with 150 µl of 1× Wash Buffer after each incubation. Signals were then detected by measuring and reading the relative fluorescence units (RFU) on a fluorescence microplate reader (ThermoFisher, Varioskan LUX, USA) at Ex/Em = 530/590 nm after adding 50 µl of Fluoro Developer Mix to each well and incubating at RT for 1–4 min away from the light. We also performed a simple calculation of the proportion (%) of m6A in the total RNA as follows:

$${\text{m}}6{\text{A}}\% = \frac{{\left( {{\text{Sample}}\;{\text{RFU}}{-} {\text{Negative}}\;{\text{Control}}\;{\text{RFU}}} \right) \div {\text{Total}}\;{\text{Sample}}\;{\text{RNA}}}}{{\left( {{\text{Positive}}\;{\text{Control}}\;{\text{RFU}}{-} {\text{Negative}}\;{\text{Control}}\;{\text{RFU}}} \right) \div {\text{Total}}\;{\text{Positive}}\;{\text{Control}}\;{\text{RNA}}}}*100\% .$$

Then, we synthesized cDNA using the PrimeScript First Strand cDNA Synthesis Kit (Takara, RR047A, Japan). Genomic DNA was first removed from the mixed 10 µl solution with 50 ng of total RNA, 2 µl of 5× gDNA eraser buffer, and 1 µl of gDNA eraser, via polymerase chain reaction (PCR) (Bio-Rad, QX200, USA) at 42 °C for 2 min. The 10 µl mixed solution was then reverse transcribed to 20 µl of cDNA by adding 1 µl of PrimeScript RT Enzyme Mix 1, 1 µl of RT Primer Mix, and 4 µl of 5× PrimeScript Buffer 2, via PCR at 37 °C for 15 min and 85 °C for 5 s. The relative RNA levels of m6A-related genes were then analyzed by quantitative real-time PCR (qRT-PCR) with the SYBR Green detection method (Takara, RR041A, Japan) and a QuantStudio 5 Real-Time PCR System (ThermoFisher, USA). The thermocycling conditions were as follows: a holding stage of 95 °C for 30 s; 40 cycles of 95 °C for 5 s and 60 °C for 30 s, followed by a melting curve stage of 95 °C for 15 s, 60 °C for 30 s, and 95 °C for 15 s. *GAPDH* was used as an internal control. The qRT-PCR reactions were performed in triplicate, and the results were analyzed using the ΔΔCT method. The primers are given in Additional file 1: Table S1.

### Statistical analysis

Statistical analyses were performed using the R (*version 4.0.2*) and GraphPad Prism (*version 9.0*) software. *P* < 0.05 was considered to indicate statistical significance. Continuous variables were calculated as medians with the standard deviations. Categorical variables were reported as a number with proportions. The *t*-test and Mann–Whitney *U* test were used to analyze the differences in continuous variables. The Chi-squared test was performed to explore differences in categorical variables.

## Results

### The identification of differentially expressed m6A RNA methylation regulators

**Fig. 1:**
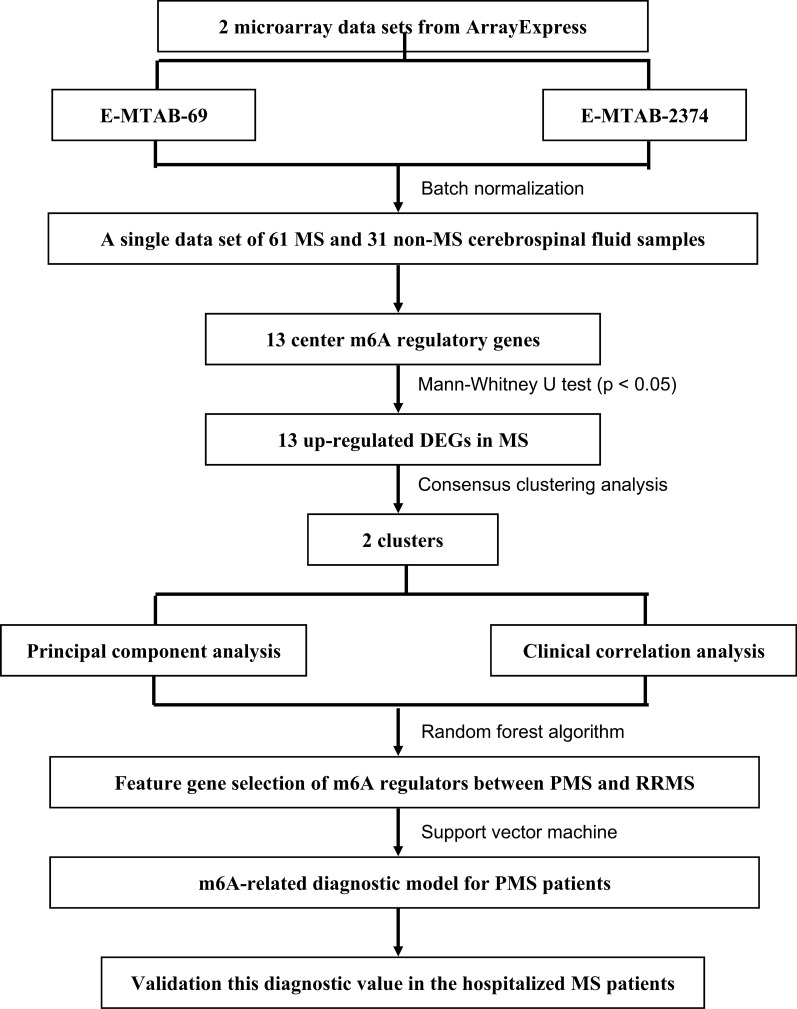
The flow chart of this study in detail

**Fig. 2:**
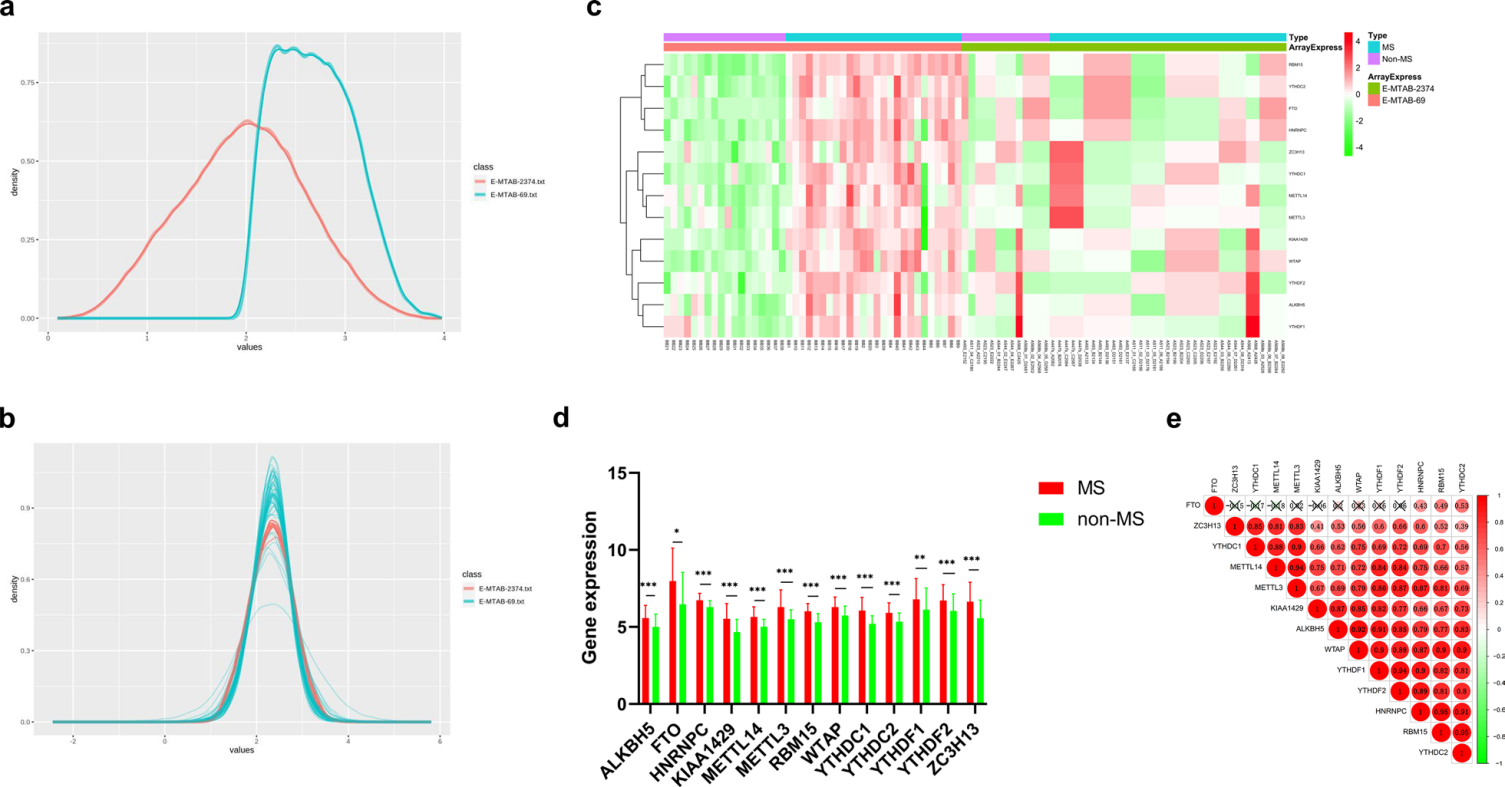
Identification of differentially expressed m6A-related genes. **a** The density plot of the two datasets before normalization. **b** The density plot of the two datasets after normalization. **c** The heat map of 13 differentially expressed m6A methylation regulators. **d** Differentially expressed m6A-related genes between MS and non-MS patients using the Mann–Whitney *U* test. (*ALKBH5*: MS vs. non-MS = 5.587 ± 0.807 vs. 5.003 ± 0.820, *p* < 0.001; *FTO*: MS vs. non-MS = 7.976 ± 2.128 vs. 6.471 ± 2.043, *p* = 0.030; *HNRNPC*: MS vs. non-MS = 6.735 ± 0.441 vs. 6.288 ± 0.410, *p* < 0.001; *KIAA1429*: MS vs. non-MS = 5.535 ± 0.970 vs. 4.664 ± 0.822, *p* < 0.001; *METTL14*: MS vs. non-MS = 5.657 ± 0.648 vs. 5.016 ± 0.476, *p* < 0.001; *METTL3*: MS vs. non-MS = 6.299 ± 1.097 vs. 5.512 ± 0.600, *p* < 0.001; *RBM15*: MS vs. non-MS = 6.023 ± 0.490 vs. 5.305 ± 0.554, *p* < 0.001; *WTAP*: MS vs. non-MS = 6.292 ± 0.644 vs. 5.742 ± 0.607, *p* < 0.001; *YTHDC1*: MS vs. non-MS = 6.068 ± 0.841 vs. 5.206 ± 0.516, *p* < 0.001; *YTHDC2*: MS vs. non-MS = 5.914 ± 0.645 vs. 5.341 ± 0.556, *p* < 0.001; *YTHDF1*: MS vs. non-MS = 6.791 ± 1.337 vs. 6.114 ± 1.393, *p* = 0.004; *YTHDF2*: MS vs. non-MS = 6.716 ± 1.016 vs. 6.045 ± 1.098, *p* < 0.001; *ZC3H13*: MS vs. non-MS = 6.645 ± 1.255 vs. 5.572 ± 1.154, *p* < 0.001). The significant levels were set at *p* < 0.05 (*), < 0.01 (**), and < 0.001 (***). **e** Correlation analysis of the relationships between different m6A-related genes

A detailed flowchart depicting this study is presented in Fig. 1. Details relating to the E-MTAB-69 and E-MTAB-2374 datasets are available in the ArrayExpress database (Additional file 1: Tables S2, S3). A single dataset was created by batch normalization for background correction and consisted of 61 MS CSF samples and 31 non-MS CSF samples (Additional file 2: Table S4). The demographic and clinical characteristics of both the MS and non-MS patients are presented in Table 1. Inter-batch differences were eliminated and the effect was confirmed via density plots that were constructed before and after batch normalization (Fig. 2a, b). Consequently, the 13 m6A RNA methylation regulators were extracted from the merged dataset (Additional file 2: Table S5). All of the 13 m6A-related genes were identified as significant DEGs between MS and non-MS patients via eBayes methods; a heat map is presented in Fig. 2c (Additional file 2: Table S6). The Mann–Whitney *U* test confirmed that the expression levels of these DEGs were significantly higher in MS patients than in non-MS patients (Fig. 2d). In addition, the Spearman correlation analysis revealed that these DEGs were positively correlated with each other except FTO (Fig. 2e).

**Table 1:** Comparison of the demographic and clinical characteristics between multiple sclerosis (MS) patients and non-MS controls in the merged dataset and in the validation group

	Datasets			Validation		
	MS (n = 61)	Non-MS (31)	*P*	MS (n = 11)	Non-MS (n = 3)	*P*
Age (years)	44.87 ± 15.66	42.71 ± 12.00	0.466	29.73 ± 10.78	42.33 ± 11.85	0.195
Sex (%)						
Female	37 (60.7%)	19 (61.3%)	0.953	8 (72.7%)	2 (66.7%)	0.837
Male	24 (39.3%)	12 (38.7%)		3 (27.3%)	1 (33.3%)	
Subtype (%)						
RRMS	32 (52.5%)	–	–	8 (72.7%)	–	–
PPMS	10 (16.4%)	–		1 (9.1%)	–	
SPMS	19 (31.1%)	–		2 (18.2%)	–	
DMDs (%)	–	–	–	3 (27.3%)	–	–
CSF testing						
OB (%)	–	–	–	1 (16.7%)	–	–
Antibody (%) (anti-AQP4, MOG, MBP)				0 (0.0%)		

In the validation group, six of the 11 MS patients underwent CSF OB testing, and only one patient found positive result. Nine of them measured related antibodies while all reported negative results

### Functional analyses of differentially expressed m6A-related genes

**Fig. 3:**
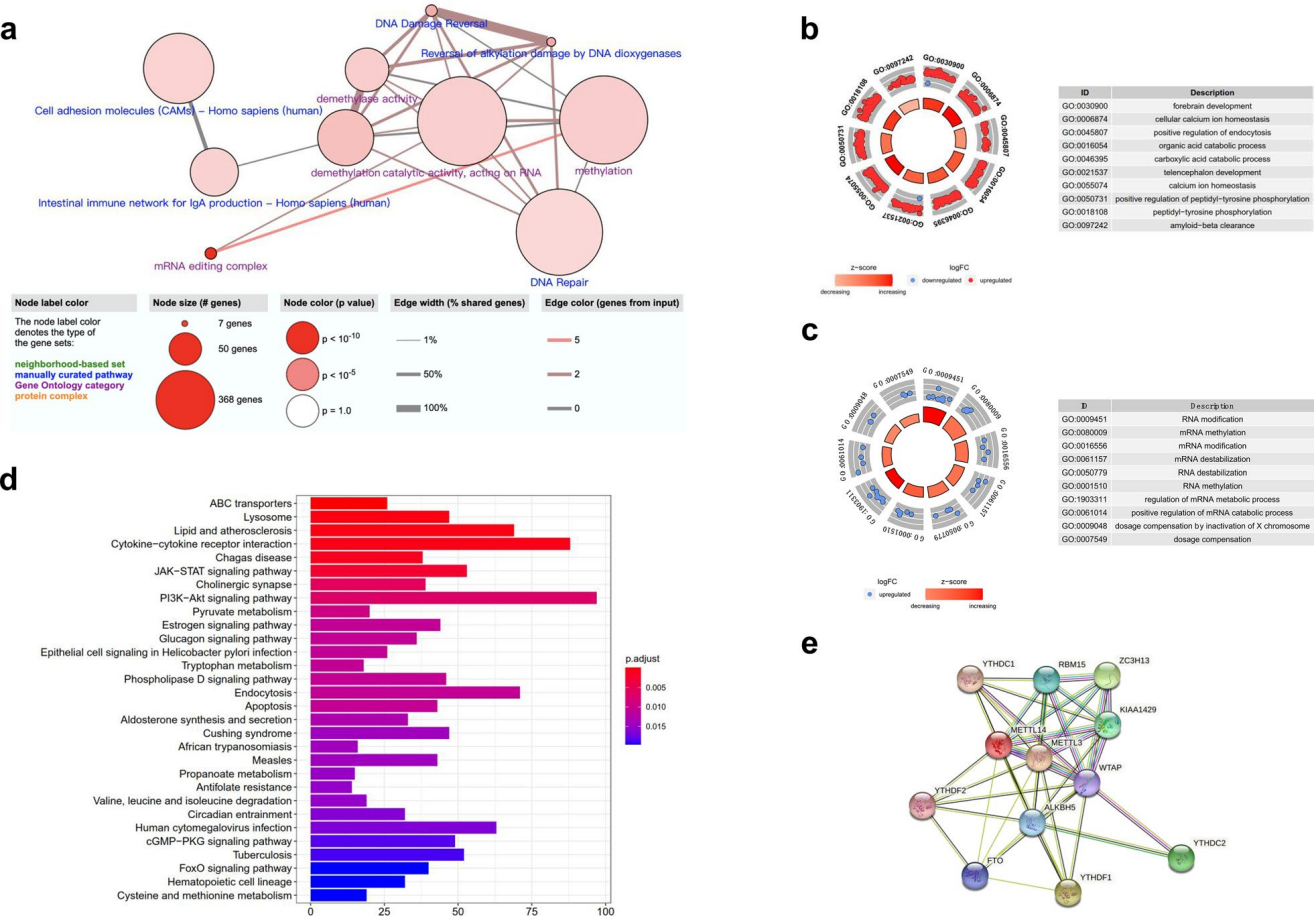
Functional annotation of the DEGs between MS and non-MS patients. **a** The integrated analysis of gene-protein interactions of top 94 DEGs and m6A-related genes. **b**, **c** The enriched GO terms of these m6A-related genes and DEGs. **d** The significant KEGG pathways of these m6A-related genes and DEGs. **e** The PPI network of these m6A-related genes with high confidence (> 0.7)

The eBayes method identified 5031 DEGs w in the merged dataset (Additional file 2: Table S7). Next, we used the ConsensusPathDB interaction database (http://consensuspathdb.org/) to construct an integrated plot to analyze the molecular function of the top 94 DEGs with a log_2_|FC| > 2 and the 13 m6A-related genes (Fig. 3a). In addition, we identified 348 significant GO items that were associated with these DEGs (Additional file 2: Table S8), mainly including cellular calcium ion homeostasis, the positive regulation of endocytosis, and the organic acid catabolic process (Fig. 3b). In addition, 157 significant GO items were shown to be associated with m6A-related genes (Additional file 2: Table S9), mainly including RNA modification, methylation, destabilization, and metabolic processes (Fig. 3c). Furthermore, 59 significant KEGG pathways were identified (Additional file 2: Table S10), including lysosomes, cytokine-cytokine receptor interaction, and the JAK-STAT signaling pathway (Fig. 3d). In addition, we used the STRING database to create a PPI network; this showed the relationships between these m6A-related regulators with high levels of high confidence (Fig. 3e).

### Non-supervision consensus clustering analysis identified two clusters of patients with MS

**Fig. 4:**
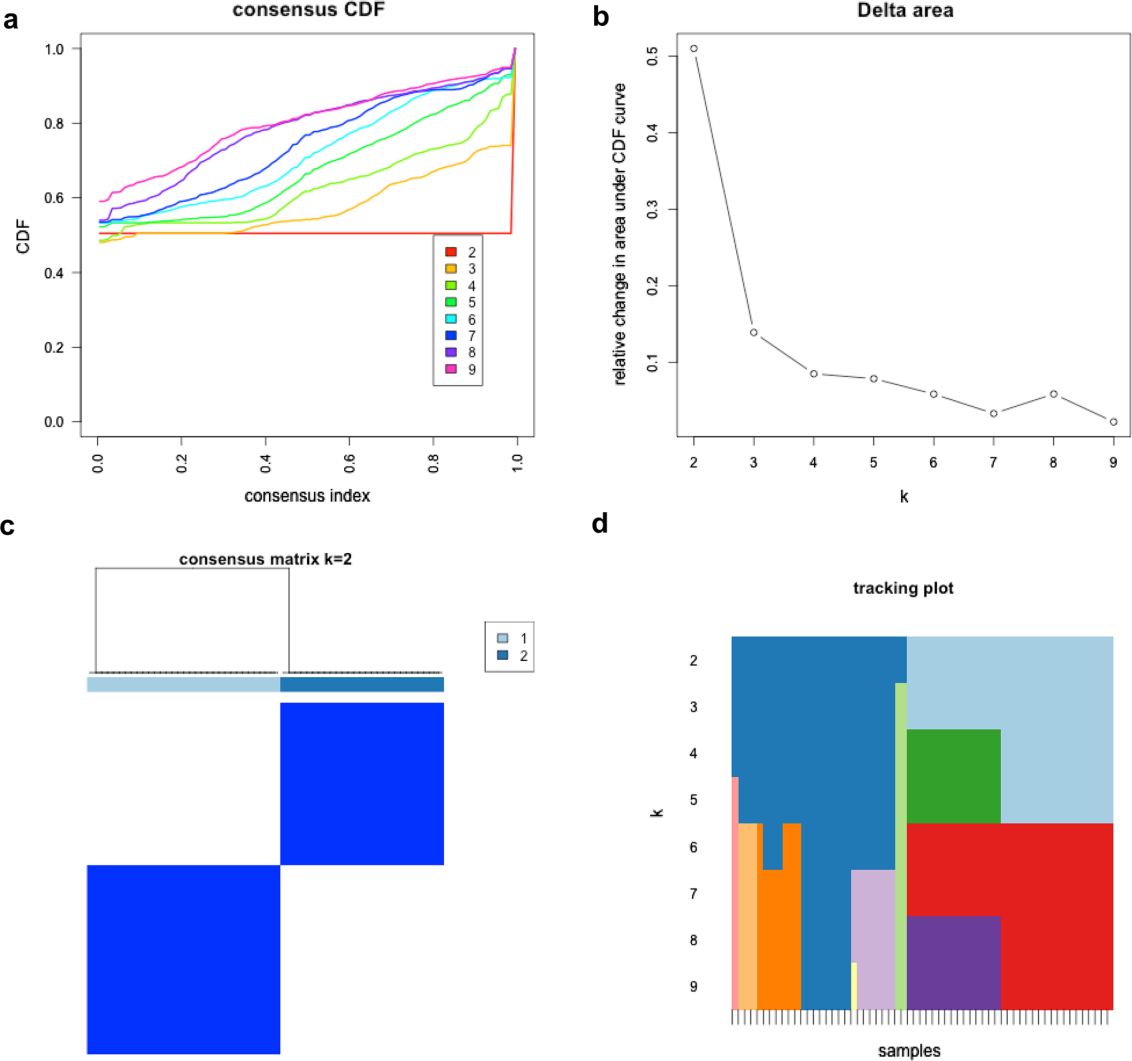
Non-supervision consensus clustering analysis of the 61 MS patients on the expression similarity of m6A-related genes. **a** The cumulative distribution function (CDF) of consensus clustering for k from 2 to 9. **b** Relative change in area under the CDF curve for k from 2 to 9. **c** The consensus clustering matrix for k = 2. **d** The tracking plot was presented to verify the principal component for k from 2 to 9

**Fig. 5:**
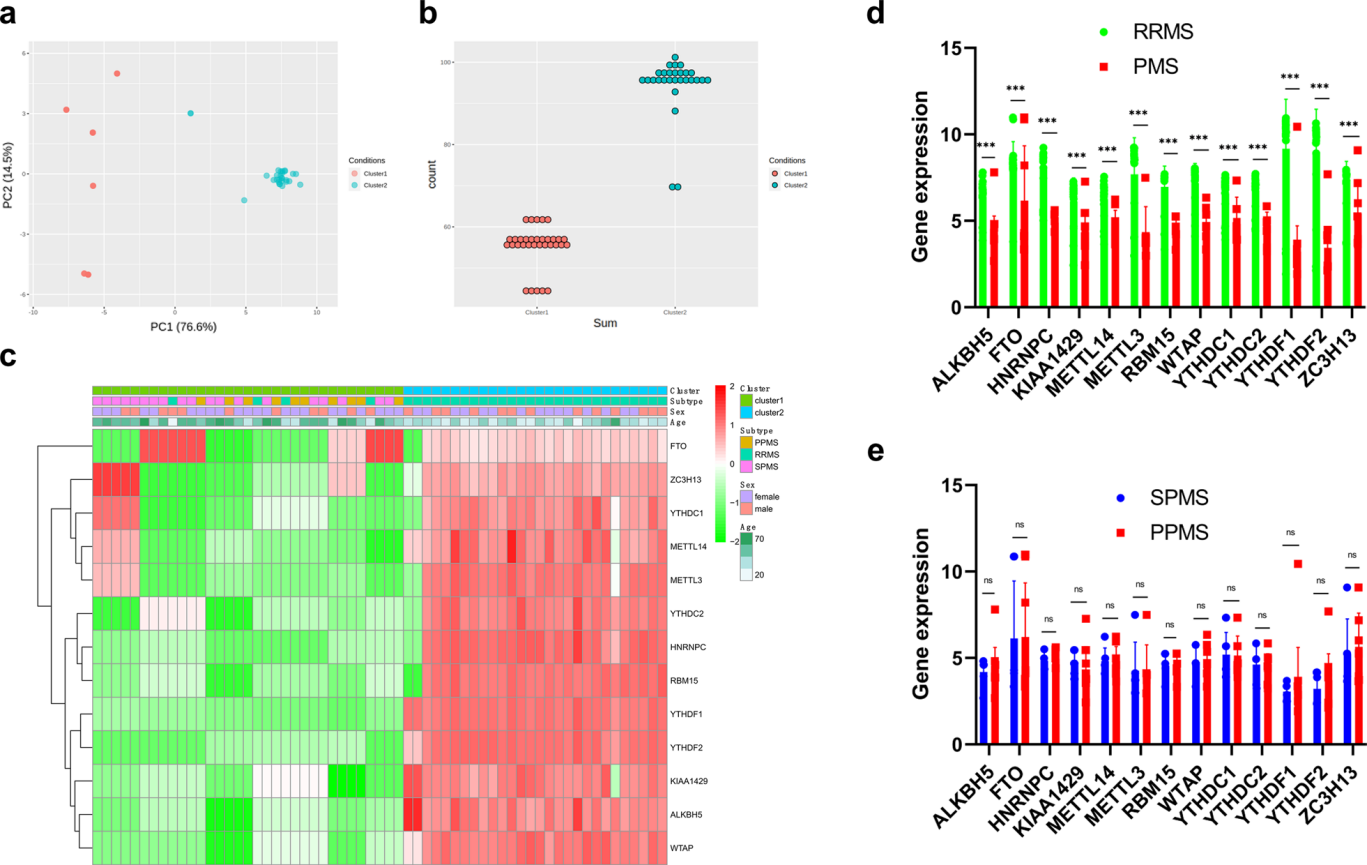
The differential clinical characteristics and gene expression between cluster 1 and cluster 2. **a** The PCA was used to verify the two distinct subgroups divided by non-supervision consensus clustering analysis of m6A-related genes. **b** The total m6A-related gene expression differences of individual patient between clusters. **c** The correlation heatmap showed a significant association between clusters and MS subtypes. **d** The gene expression differences of each m6A-related gene between PMS patients and RRMS patients using the Mann–Whitney *U* test. (*ALKBH5*: RRMS vs. PMS = 6.378 ± 0.995 vs. 4.259 ± 1.000, *p* < 0.001; *FTO*: RRMS vs. PMS = 8.411 ± 1.153 vs. 6.172 ± 3.121, *p* < 0.001; *HNRNPC*: RRMS vs. PMS = 8.006 ± 1.277 vs. 4.897 ± 0.494, *p* < 0.001; *KIAA1429*: RRMS vs. PMS = 6.132 ± 1.322 vs. 4.494 ± 1.000, *p* < 0.001; *METTL14*: RRMS vs. PMS = 6.212 ± 1.026 vs. 4.805 ± 0.787, *p* < 0.001; *METTL3*: RRMS vs. PMS = 7.688 ± 2.088 vs. 4.267 ± 1.530, *p* < 0.001; *RBM15*: RRMS vs. PMS = 6.963 ± 1.186 vs. 4.477 ± 0.671, *p* < 0.001; *WTAP*: RRMS vs. PMS = 7.177 ± 1.119 vs. 4.810 ± 0.874, *p* < 0.001; *YTHDC1*: RRMS vs. PMS = 6.563 ± 1.085 vs. 5.167 ± 1.185, *p* < 0.001; *YTHDC2*: RRMS vs. PMS = 6.749 ± 0.944 vs. 4.533 ± 0.951, *p* < 0.001; *YTHDF1*: RRMS vs. PMS = 9.171 ± 2.812 vs. 3.223 ± 1.457, *p* < 0.001; *YTHDF2*: RRMS vs. PMS = 8.856 ± 2.558 vs. 3.445 ± 1.191, *p* < 0.001; *ZC3H13*: RRMS vs. PMS = 7.239 ± 1.191 vs. 5.413 ± 1.938, *p* < 0.001). **e** The gene expression differences of each m6A-related gene between SPMS patients and PPMS patients using the Mann–Whitney *U* test. (*ALKBH5*: SPMS vs. PPMS = 4.189 ± 0.737 vs. 4.346 ± 1.362, *p* = 0.687; *FTO*: SPMS vs. PPMS = 6.136 ± 3.202 vs. 6.217 ± 2.911, *p* = 0.947; *HNRNPC*: SPMS vs. PPMS = 6.136 ± 0.507 vs. 6.217 ± 0.467, *p* = 0.588; *KIAA1429*: SPMS vs. PPMS = 4.643 ± 0.823 vs. 4.311 ± 1.268, *p* = 0.391; *METTL14*: SPMS vs. PPMS = 4.815 ± 0.737 vs. 4.792 ± 0.852, *p* = 0.941; *METTL3*: SPMS vs. PPMS = 4.272 ± 1.501 vs. 4.260 ± 1.564, *p* = 0.984; *RBM15*: SPMS vs. PPMS = 4.551 ± 0.633 vs. 4.387 ± 0.700, *p* = 0.531; *WTAP*: SPMS vs. PPMS = 4.767 ± 0.821 vs. 4.862 ± 0.965, *p* = 0.779; *YTHDC1*: SPMS vs. PPMS = 5.189 ± 1.183 vs. 5.139 ± 1.187, *p* = 0.915; *YTHDC2*: SPMS vs. PPMS = 4.625 ± 0.974 vs. 4.421 ± 0.863, *p* = 0.583; *YTHDF1*: SPMS vs. PPMS = 3.062 ± 0.504 vs. 3.421 ± 2.311, *p* = 0.526; *YTHDF2*: SPMS vs. PPMS = 3.220 ± 0.890 vs. 3.721 ± 1.568, *p* = 0.276; *ZC3H13*: SPMS vs. PPMS = 5.228 ± 1.899 vs. 5.641 ± 1.993, *p* = 0.584). The significant levels were set at *p* < 0.05 (*), < 0.01 (**), and < 0.001 (***)

The total gene expression levels of these 13 m6A RNA methylation regulators were used to classify the 61 MS patients into different clusters on the basis of non-supervision consensus clustering analysis. When the clustering index “k” increased from 2 to 9, k = 2 was demonstrated to be the optimal point with which to identify the largest differences and the smallest interferences between clusters (Fig. 4). Consequently, the 61 MS patients were automatically classified into two clusters: cluster 1 and cluster 2. Next, we used a PCA plot was used to verify the effect of classification, as shown in Fig. 5a. A count plot was used to confirm the different quantification of m6A RNA methylation between clusters (Fig. 5b). We also plotted a heatmap to express the differences in gene expression of these 13 m6A-related genes and together with demographic and clinical characteristics between the clusters (Fig. 5c). Interestingly, all of the patients with PMS were classified into cluster 1 while most of the patients with RRMS were divided into cluster 2; this suggested that the dynamic m6A RNA modification in CSF might be a diagnostic biomarker with which to distinguish PMS from RRMS. Differences in the expression of these m6A-related genes between the PMS and RRMS subgroups were statistically significant, as determined by the Mann–Whitney *U* test (Fig. 5d); there were no statistical differences between the SPMS and PPMS groups (Fig. 5e).

### The identification and evaluation of an m6A-related diagnostic gene signature

**Fig. 6:**
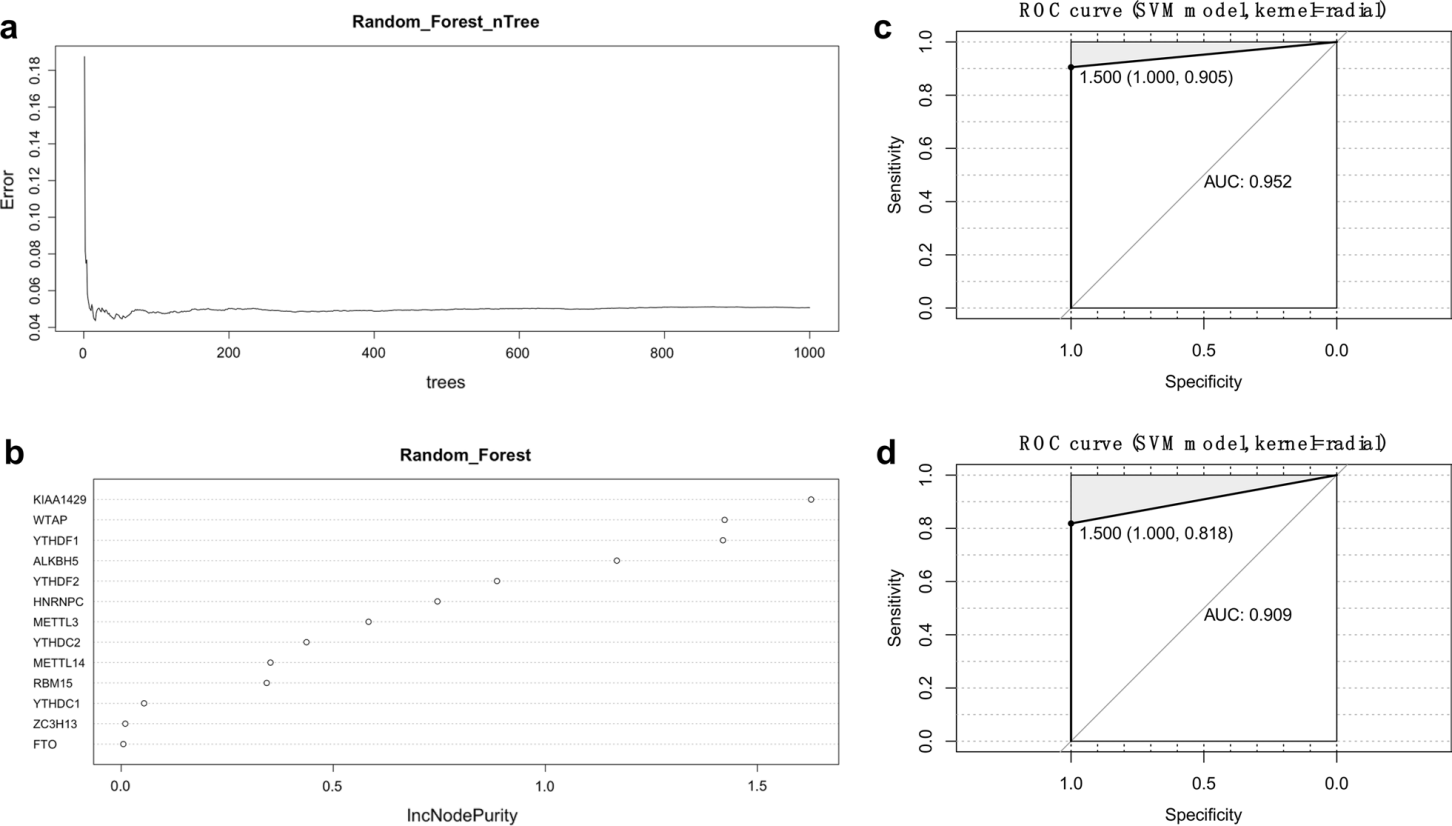
The m6A-related feature gene selection and the diagnostic gene signature construction. **a** The random forest algorithm revealed that the error is small and stable after 400 nTree in the training set. **b** Eight feature genes were selected according to the cutoff value of 0.4, including *KIAA1429*, *WTAP*, *YTHDF1*, *ALKBH5*, *YTHDF2*, *HNRNPC*, *METTL3*, and *YTHDC2*. **c** The ROC curve for assessing the performance of this diagnostic gene signature in the training set. **d** The ROC curve for assessing the performance of this diagnostic gene signature in the test set

In this study, we used the Spearman’s correlation analyses to investigate the effect of influence of age and gender on the expression of key genes. There was no significant correlation between gender and any of the m6A-related genes. However, age exhibited a negative relationship with ALKBH5, HNRNPC, KIAA1429, METTL14, METTL3, YTHDC2, YTHDF1, YTHDF2, and WTAP (Additional file 3: Fig. S1). Indeed, in order to eliminate the effects of this factor, we randomly divided these MS patients into a training set for model development (2/3, n = 41) and a test set for model validation (1/3, n = 20). There were no significant differences in terms of demographic and clinical characteristics when compared between the training set (Additional file 2: Table S11) and the test set (Additional file 2: Table S12), as shown in Table 2. The RF algorithm was applied to the training set to identify key parameters with the nTree set to 1000; this analysis revealed that the error was small and stable after the 400 nTree (Fig. 6a). Then, we calculated and ranked the importance of the 13 m6A-related genes (Fig. 6b). A total of eight feature genes were selected using a cut-off value of 0.4: *KIAA1429*, *WTAP*, *YTHDF1*, *ALKBH5*, *YTHDF2*, *HNRNPC*, *METTL3*, and *YTHDC2*. Subsequently, an m6A-related diagnostic gene signature was constructed in the training set of data; for this, we used the SVM method with a type of C-classification and a kernel of radial. Next, we validated the diagnostic model in the test set. ROC curve analysis yielded an AUC of 0.952 in the training set and an AUC of 0.909 in the test set, thus demonstrating that the diagnostic gene signature performed well (Fig. 6c, d). Next, we applied PCA analysis to evaluate the performance of the SVM classifiers based on the correlation-based distances. This diagnostic gene signature was successfully able to distinguish PMS cases from RRMS in both the training and test sets (Additional file 4: Fig. S2).

**Table 2:** Comparison of the demographic and clinical characteristics between training set and test set

	Training (n = 41)	Test (n = 20)	*P*
Age (years)	45.0 ± 14.5	44.7 ± 18.2	0.954
Female (%)	26 (63.4%)	11 (55%)	0.528
Diagnosis (%)			
RRMS	21 (51.2%)	11 (55.0%)	0.959
SPMS	13 (31.7%)	6 (30.0%)	
PPMS	7 (17.1%)	3 (15.0%)

### External validation confirmed the performance of the diagnostic model in MS and non-MS patients

**Fig. 7:**
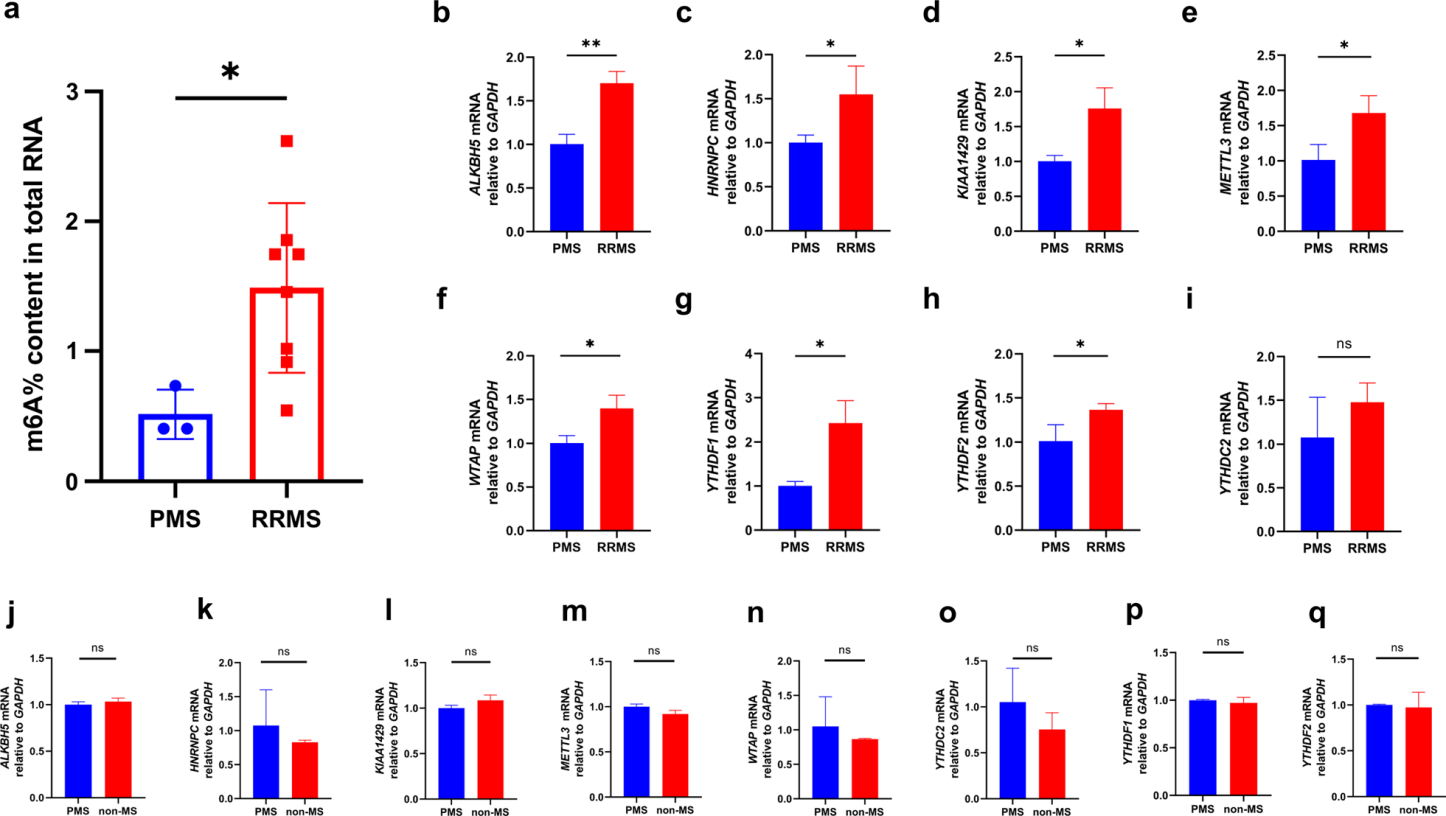
The total m6A level and qRT-PCR validation of the m6A-related feature genes in patients with MS. **a** The total m6A RNA methylation context of total RNA between PMS patients and RRMS patients (PMS vs. RRMS = 0.515 ± 0.154% vs. 1.488 ± 0.611%, *p* = 0.036). **b**–**i** The gene expression of the feature genes *ALKBH5*, *HNRNPC*, *KIAA1429*, *METTL3*, *WTAP*, *YTHDF1*, *YTHDF2*, and *YTHDC2* between PMS patients and RRMS patients (*ALKBH5*: PMS vs. RRMS = 1.004 ± 0.091 vs. 1.702 ± 0.110, *p* = 0.002; *HNRNPC*: PMS vs. RRMS = 1.002 ± 0.070 vs. 1.549 ± 0.262, *p* = 0.046; *KIAA1429*: PMS vs. RRMS = 1.003 ± 0.068 vs. 1.760 ± 0.242, *p* = 0.013; *METTL3*: PMS vs. RRMS = 1.015 ± 0.178 vs. 1.680 ± 0.202, *p* = 0.025; *WTAP*: PMS vs. RRMS = 1.002 ± 0.072 vs. 1.399 ± 0.123, *p* = 0.017; *YTHDF1*: PMS vs. RRMS = 1.003 ± 0.083 vs. 2.432 ± 0.407, *p* = 0.008; *YTHDF2*: PMS vs. RRMS = 1.012 ± 0.153 vs. 1.364 ± 0.057, *p* = 0.038; *YTHDC2*: PMS vs. RRMS = 1.078 ± 0.372 vs. 1.478 ± 0.180, *p* = 0.243). **j**–**q** The gene expression of the feature genes *ALKBH5*, *HNRNPC*, *KIAA1429*, *METTL3*, *WTAP*, *YTHDF1*, *YTHDF2*, and *YTHDC2* between PMS patients and non-MS patients (*ALKBH5*: PMS vs. non-MS = 1.000 ± 0.026 vs. 1.034 ± 0.030, *p* = 0.295; *HNRNPC*: PMS vs. non-MS = 1.075 ± 0.429 vs. 0.830 ± 0.024, *p* = 0.465; *KIAA1429*: PMS vs. non-MS = 1.000 ± 0.025 vs. 1.085 ± 0.048, *p* = 0.092; *METTL3*: PMS vs. non-MS = 1.000 ± 0.024 vs. 0.920 ± 0.034, *p* = 0.005; *WTAP*: PMS vs. non-MS = 1.052 ± 0.351 vs. 0.866 ± 0.010, *p* = 0.495; *YTHDC2*: PMS vs. non-MS = 1.051 ± 0.301 vs. 0.753 ± 0.149, *p* = 0.277; *YTHDF1*: PMS vs. non-MS = 1.000 ± 0.007 vs. 0.972 ± 0.047, *p* = 0.457; *YTHDF2*: PMS vs. non-MS = 1.000 ± 0.006 vs. 0.973 ± 0.135, *p* = 0.790). The significant levels were set at *p* < 0.05 (*), < 0.01 (**), and < 0.001 (***)

A total of 20 patients diagnosed with MS underwent lumbar puncture in our hospital between July 2020 and December 2020. We excluded CSF samples from nine patients because of very low total RNA concentration and/or poor RNA quality. Of the 11 remaining patients, one had PPMS, two had SPMS, and eight had RRMS. The clinical and demographic characteristics of the external validation cohort are presented in Table 1. The quantification of total m6A RNA methylation was evaluated by reading individual RFU data with a fluorescence microplate reader. The proportion (%) of m6A in total RNA was calculated and is presented in Fig. 7a; this data confirmed that the total levels of m6A RNA methylation were relatively lower in patients with PMS than those with RRMS. In addition, seven of the eight feature m6A-related genes were verified by qRT-PCR (Fig. 7b–h). RRMS patients exhibited higher expression levels of *YTHDC2* than those with PMS, although this was not statistically significant (Fig. 7i). Nevertheless, we also recruited three patients who had been diagnosed with neurodegenerative disorders [including one case of PD and two cases of amyotrophic lateral sclerosis (ALS)] as non-MS controls between May 2021 and June 2021. These feature genes were also tested; however, the expression levels of these genes did not differ significantly when compared between PMS patients and non-MS patients (Fig. 7j–q). In short, this diagnostic model seems to be a potential tool to help to distinguish MS subtypes.

## Discussion

In this study, we found that 13 central m6A RNA methylation regulators were all upregulated in the CSF of MS patients when compared with non-MS patients. Non-supervision consensus clustering analysis further identified two clusters of MS samples according to different m6A RNA modification levels; these two clusters were significantly associated with MS subtypes. The RF algorithm and SVM methodology successfully identified an m6A-related diagnostic gene signature. Further evaluation, using both training and test sets, showed that this diagnostic model exhibited well performance. In addition, we also quantified total m6A RNA methylation levels and carried out qRT-PCR to verify these findings in a small external validation cohort that included 11 patients with MS and 3 non-MS patients.

To our knowledge, this is the first report to describe m6A RNA methylation changes in MS. Previous studies have only demonstrated elevated levels of DNA methylation and the dynamic changes of differentially methylated regions in MS patients; these changes were significantly and positively associated with the expanded disability status scale (EDSS) score and progression index (PI) [17–19]. Previous research demonstrated an association between pathological MS lesions, DNA methyltransferase, and hypermethylated oligodendrocyte survival genes, thus suggesting that changes in methylation could represent a potential target that can accelerate the course of MS disease [20–22]. In contrast, Singhal et al. [23] found that betaine, a methyl donor, played a neuroprotective role in the cuprizone mouse model of MS by increasing the rate of methylation and by preventing mitochondrial impairment. Consistent with this result, we observed reduced levels of methylation in PMS patients when compared with RRMS patients, thus indicating that elevated methylation levels might provide neuroprotection for patients with MS. A previous study showed that fumaric acid esters exhibit a direct and dose-dependent effect on hypermethylation to protect MS patients from relapse [24]. Another study demonstrated that global methylation levels were negatively correlated with treatment duration in MS patients who were administered with IFN-β, thus suggesting that total methylation levels are a potential and reliable biomarker of the clinical response to DMDs [25]. In addition, cigarette smoking is understood to promote the disease process in MS patients via DNA methylation [26]. Increased body weight may also alleviate the course of disease by regulating ceramide-induced anti-proliferative gene methylation to modulate the infiltration of monocytes [27]. Consequently, these previous studies have highlighted the possible importance of methylation modification in the pathogenesis of MS.

In this study, we developed an m6A-related diagnostic gene signature that would allow us to distinguish PMS from RRMS; most of these feature genes were m6A readers and writers (with relatively lower gene expression levels in PMS). These m6A readers and writers have previously been reported to act as key epigenetic factors in neurodevelopment, synaptogenesis, axon guidance, and neural repair [28]. Knockout of the m6A reader *METTL3* is known to inhibit neural proliferation and maturation while loss of the m6A reader *METTL14* is known to reduce the regeneration of functional axons [29, 30]. Mutation of the m6A reader *YTHDF1* is known to delay pre-crossing axonal guidance by influencing the expression levels of Robo3.1 mRNA [31]. In addition, recent studies have demonstrated that the dysregulation of RNA methylation is associated with multiple biological processes in neurodegenerative diseases. For example, *METTL3* was shown to be upregulated in brain tissues and positively correlated with the concentration of Tau protein [8, 28]. Levels of the m6A eraser *FTO* were shown to be significantly reduced in AD patients while risky genetic variations were correlated with approximately 8% and 12% of brain volume deficits in the frontal and occipital lobes of patients with AD, respectively [29, 30]. In addition, Hess et al. [31] found that the inactivation of *FTO* had a negative impact on the dopamine receptors in a mouse model of PD, thus leading to a reduction in quinpirole-mediated motion function and increased levels of adenosine methylation in the *FTO*-deficient mice, thus indicating that m6A-related genes regulated the RNA methylation of hub genes to control the dopamine transmission in PD [31]. Collectively, these studies provided reliable evidence to prove that alterations in m6A RNA methylation are highly associated with neurodegenerative disorders. However, in contrast to the scenario observed for AD and PD, PMS patients exhibited methylation levels that were lower than those with RRMS. Inflammatory and demyelination are known to cause several pathological hallmarks, including axonal loss, gray matter pathology, and immune cell infiltrations in RRMS patients; however, PMS patients do not exhibit an obviously active immunization status. Current evidences proves that the methylation of m6A RNA is highly associated with immune recognition, the activation of innate and adaptive immune responses, and cell fate decisions [32]. Thus, the extent of total m6A RNA methylation was relatively higher in patients with RRMS. In addition, because of the ethical considerations related to the use of lumbar puncture on healthy controls, we were only able to obtain control CSF samples from patients with neurodegenerative disorders. Consequently, there were no obvious differences between controls and PMS patients in the external validation set (which only featured a small number of samples), thus suggesting that the expression levels of m6A-related genes were similar.

PMS is an uncommon and severe subtype of MS that leads to a gradual decline and irreversible disabilities without appropriate treatment. It is important to diagnose PMS at disease onset; however, this represents a significant challenge. Current diagnostic criteria can usually extend the course of disease, as determined by retrospective history. Neurofilament light chain (NfL) was previously reported to be a reliable biomarker with which to diagnose RRMS [33]. However, levels of NfL are also known to be increased in a variety of neurological diseases associated with axonal injury or degeneration, including inflammatory, neurodegenerative, traumatic, and cerebrovascular diseases [34]. Therefore, NfL cannot be used for the specific diagnosis of RRMS. In contrast, several studies have reported significant differences in NfL levels when compared between PMS and RRMS; thus, NfL may have the potential to predict conversion from RRMS to SPMS [35, 36]. Although NfL may exhibit diagnostic and prognostic value for PMS, this hypothesis should now be tested with a large sample size [37–39]. In addition, the integrated analysis of 11 radiomics, metabolomics, and proteomic characteristics was shown to lead to an earlier diagnosis of SPMS in a previous limited cohort of patients, although the high cost and stringent conditions required rendered this analysis difficult to apply for PPMS [40]. Presently, we know very little about the true diagnostic value of biological biomarkers for PMS; as such, we do not yet have an efficient tool with which to specifically diagnose PMS. Therefore, personalized therapeutic advice for preventing neurological deterioration in patients with PMS is not yet evidence-based. In the present study, we identified possible diagnostic biomarkers for PMS from CSF samples based on m6A regulatory genes. Validation tests demonstrated that this gene signature showed good performance for distinguishing between PMS and RRMS.

Our study has some limitations that should be considered. First, the complete clinical characteristics of MS patients were not available in the original datasets; PMS patients usually have a higher EDSS, and a shorter disease duration, than RRMS patients. Furthermore, DMDs have been shown to be beneficial for prolonged transitional disease duration in SPMS [41]. Thus, future research should consider the precise relationships between these observations and the expression of m6A-related genes. Second, this m6A-related gene signature was only verified in a small cohort. A randomized control study, with a larger sample size, should now be conducted to validate our findings. Third, lumbar puncture is an invasive assessment for MS patients. Consequently, future research should investigate the expression of these biomarkers in whole and/or peripheral blood mono-nucleic cells (PBMCs). Finally, the therapeutic effect of DMDs on the methylation of m6A RNA methylation should be assessed in order to identify effective targets.

## Conclusions

In conclusion, this preliminary study suggested that the dynamic modification of m6A RNA methylation is involved in the progression of MS and is likely to represent a novel CSF diagnostic biomarker for distinguishing PMS from RRMS at early disease onset.

## Supplementary Information

**Additional file 1: Table S1.** The primers of the 13 m6A-related genes. **Table S2.** The original gene expression matrix of E-MTAB-69 from ArrayExpress database. **Table S3.** The original gene expression matrix of E-MTAB-2374 from ArrayExpress database.

**Additional file 2: Table S4.** The merged gene expression matrix from E-MTAB-69 and E-MTAB-2374 via batch-normalization. **Table S5.** The 13 m6A RNA methylation regulators in the merged data set. **Table S6.** The differentially expressed analysis of the 13 m6A RNA methylation regulators between MS patients and non-MS controls. **Table S7.** Identification of differentially expressed genes (DEGs) between MS patients and non-MS controls. **Table S8.** The gene ontology (GO) analysis of the DEGs. **Table S9.** The gene ontology (GO) analysis of the 13 m6A RNA methylation regulators. **Table S10.** The Kyoto Encyclopedia of Genes and Genomes (KEGG) pathways of the DEGs. **Table S11.** The clinical characteristic in the training set (n = 41).

**Additional file 3: Figure S1.** Correlation analysis of the relationships between different m6A-related genes and demographic characteristics (age or sex).

**Additional file 4: Figure S2.** The PCA analysis was applied to evaluation of the performance of SVM classifiers based on the correlation-based distances in the training set and in the test set.

## Data Availability

The datasets in this study can be found in the ArrayExpress database.

## Abbreviations

MS: Multiple sclerosis
CNS: Central nervous system
RRMS: Relapsing–remitting multiple sclerosis
SPMS: Secondary progressive multiple sclerosis
PPMS: Primary progressive multiple sclerosis
PMS: Progressive multiple sclerosis
CSF: Cerebrospinal fluid
DMDs: Disease modifying drugs
m6A: *N*6-Methyladenosine
AD: Alzheimer’s disease
PD: Parkinson’s disease
OB: Oligoclonal bands
GEO: Gene Expression Omnibus
RMA: Robust multi-array average
DEGs: Differentially expressed genes
eBayes: Empirical Bayes
FC: Fold change
FDR: False discovery rate
GO: Gene Oncology
KEGG: Kyoto Encyclopedia of Genes and Genomes
PPI: Protein–protein interaction
STRING: Search Tool for the Retrieval of Interacting Genes
PCA: Principal component analysis
RF: Random forest
SVM: Support vector machine
ROC: Receiver operating characteristic
RT: Room temperature
RFU: Relative fluorescence units
PCR: Polymerase chain reaction
qRT-PCR: Quantitative real-time PCR
ALS: Amyotrophic lateral sclerosis
EDSS: Expanded disability status scale
PI: Progression index
NfL: Neurofilament light chain
CDF: Cumulative distribution function
PBMCs: Peripheral blood mono-nucleic cells

## References

[1] Thompson AJ, Baranzini SE, Geurts J (2018). Multiple sclerosis. Lancet.

[2] Rovaris M, Confavreux C, Furlan R (2006). Secondary progressive multiple sclerosis: current knowledge and future challenges. Lancet Neurol.

[3] Miller DH, Leary SM (2007). Primary-progressive multiple sclerosis. Lancet Neurol.

[4] Thompson AJ, Banwell BL, Barkhof F (2018). Diagnosis of multiple sclerosis: 2017 revisions of the McDonald criteria. Lancet Neurol.

[5] Feinstein A, Freeman J, Lo AC (2015). Treatment of progressive multiple sclerosis: what works, what does not, and what is needed. Lancet Neurol.

[6] Shulman Z, Stern-Ginossar N (2020). The RNA modification *N*^6^-methyladenosine as a novel regulator of the immune system. Nat Immunol.

[7] Faissner S, Plemel JR, Gold R (2019). Progressive multiple sclerosis: from pathophysiology to therapeutic strategies. Nat Rev Drug Discov.

[8] Han M, Liu Z, Xu Y (2020). Abnormality of m6A mRNA methylation is involved in Alzheimer’s disease. Front Neurosci.

[9] Qin L, Min S, Shu L (2020). Genetic analysis of *N*6-methyladenosine modification genes in Parkinson’s disease. Neurobiol Aging.

[10] Mueller AM, Yoon BH, Sadiq SA (2014). Inhibition of hyaluronan synthesis protects against central nervous system (CNS) autoimmunity and increases CXCL12 expression in the inflamed CNS. J Biol Chem.

[11] Müller AM, Jun E, Conlon H (2012). Cerebrospinal hepatocyte growth factor levels correlate negatively with disease activity in multiple sclerosis. J Neuroimmunol.

[12] Wu S, Li G, Deng L (2019). L1-norm batch normalization for efficient training of deep neural networks. IEEE Trans Neural Netw Learn Syst.

[13] Yang Y, Hsu PJ, Chen YS (2018). Dynamic transcriptomic m^6^A decoration: writers, erasers, readers and functions in RNA metabolism. Cell Res.

[14] The Gene Ontology Consortium (2017). Expansion of the Gene Ontology knowledgebase and resources. Nucleic Acids Res.

[15] Kanehisa M, Sato Y, Kawashima M (2016). KEGG as a reference resource for gene and protein annotation. Nucleic Acids Res.

[16] Szklarczyk D, Gable AL, Lyon D (2019). STRING v11: protein–protein association networks with increased coverage, supporting functional discovery in genome-wide experimental datasets. Nucleic Acids Res.

[17] Fernandes SJ, Morikawa H, Ewing E (2019). Non-parametric combination analysis of multiple data types enables detection of novel regulatory mechanisms in T cells of multiple sclerosis patients. Sci Rep.

[18] Maltby VE, Lea RA, Burnard S (2020). Epigenetic differences at the HTR2A locus in progressive multiple sclerosis patients. Sci Rep.

[19] Gharibi T, Hosseini A, Marofi F (2019). IL-21 and IL-21-producing T cells are involved in multiple sclerosis severity and progression [published correction appears in Immunol Lett. 2021 Jan 15]. Immunol Lett.

[20] Mo XB, Lei SF, Qian QY (2019). Integrative analysis revealed potential causal genetic and epigenetic factors for multiple sclerosis. J Neurol.

[21] Chomyk AM, Volsko C, Tripathi A (2017). DNA methylation in demyelinated multiple sclerosis hippocampus. Sci Rep.

[22] Huynh JL, Garg P, Thin TH (2014). Epigenome-wide differences in pathology-free regions of multiple sclerosis-affected brains. Nat Neurosci.

[23] Singhal NK, Sternbach S, Fleming S (2020). Betaine restores epigenetic control and supports neuronal mitochondria in the cuprizone mouse model of multiple sclerosis. Epigenetics.

[24] Ntranos A, Ntranos V, Bonnefil V (2019). Fumarates target the metabolic-epigenetic interplay of brain-homing T cells in multiple sclerosis. Brain.

[25] Pinto-Medel MJ, Oliver-Martos B, Urbaneja-Romero P (2017). Global methylation correlates with clinical status in multiple sclerosis patients in the first year of IFNbeta treatment. Sci Rep.

[26] Ringh MV, Hagemann-Jensen M, Needhamsen M (2020). Methylome and transcriptome signature of bronchoalveolar cells from multiple sclerosis patients in relation to smoking. Multiple Scler J.

[27] Castro K, Ntranos A, Amatruda M (2019). Body mass index in multiple sclerosis modulates ceramide-induced DNA methylation and disease course. EBioMedicine.

[28] Dermentzaki G, Lotti F (2020). New insights on the role of *N*^6^-methyladenosine RNA methylation in the physiology and pathology of the nervous system. Front Mol Biosci.

[29] Chen X, Yu C, Guo M (2019). Down-regulation of m6A mRNA methylation is involved in dopaminergic neuronal death. ACS Chem Neurosci.

[30] Weng YL, Wang X, An R (2018). Epitranscriptomic m^6^A regulation of axon regeneration in the adult mammalian nervous system. Neuron.

[31] Zhuang M, Li X, Zhu J (2019). The m6A reader YTHDF1 regulates axon guidance through translational control of Robo3.1 expression. Nucleic Acids Res.

[32] Huang H, Camats-Perna J, Medeiros R (2020). Altered expression of the m6A methyltransferase METTL3 in Alzheimer’s disease. eNeuro.

[33] Ho AJ, Stein JL, Hua X (2010). A commonly carried allele of the obesity-related FTO gene is associated with reduced brain volume in the healthy elderly. Proc Natl Acad Sci USA.

[34] Reitz C, Tosto G, Mayeux R (2012). Genetic variants in the fat and obesity associated (FTO) gene and risk of Alzheimer’s disease. PLoS ONE.

[35] Hess ME, Hess S, Meyer KD (2013). The fat mass and obesity associated gene (Fto) regulates activity of the dopaminergic midbrain circuitry. Nat Neurosci.

[36] Ferrazzano G, Crisafulli SG, Baione V (2020). Early diagnosis of secondary progressive multiple sclerosis: focus on fluid and neurophysiological biomarkers. J Neurol.

[37] Gaetani L, Blennow K, Calabresi P, Di Filippo M, Parnetti L, Zetterberg H (2019). Neurofilament light chain as a biomarker in neurological disorders. J Neurol Neurosurg Psychiatry.

[38] Bhan A, Jacobsen C, Myhr KM, Dalen I, Lode K, Farbu E (2018). Neurofilaments and 10-year follow-up in multiple sclerosis. Multiple Scler J.

[39] Salzer J, Svenningsson A, Sundström P (2010). Neurofilament light as a prognostic marker in multiple sclerosis. Multiple Scler J.

[40] Herman S, Khoonsari PE, Tolf A (2018). Integration of magnetic resonance imaging and protein and metabolite CSF measurements to enable early diagnosis of secondary progressive multiple sclerosis. Theranostics.

[41] Inojosa H, Proschmann U, Akgün K, Ziemssen T (2021). A focus on secondary progressive multiple sclerosis (SPMS): challenges in diagnosis and definition. J Neurol.

