# Supplementary figures and images for: N6-Methyladenosine RNA modification in cerebrospinal fluid as a novel potential diagnostic biomarker for progressive multiple sclerosis
Source: J Transl Med. 2021 Jul 22;19:316. doi: 10.1186/s12967-021-02981-5 (PMC8296732; doi:10.1186/s12967-021-02981-5)

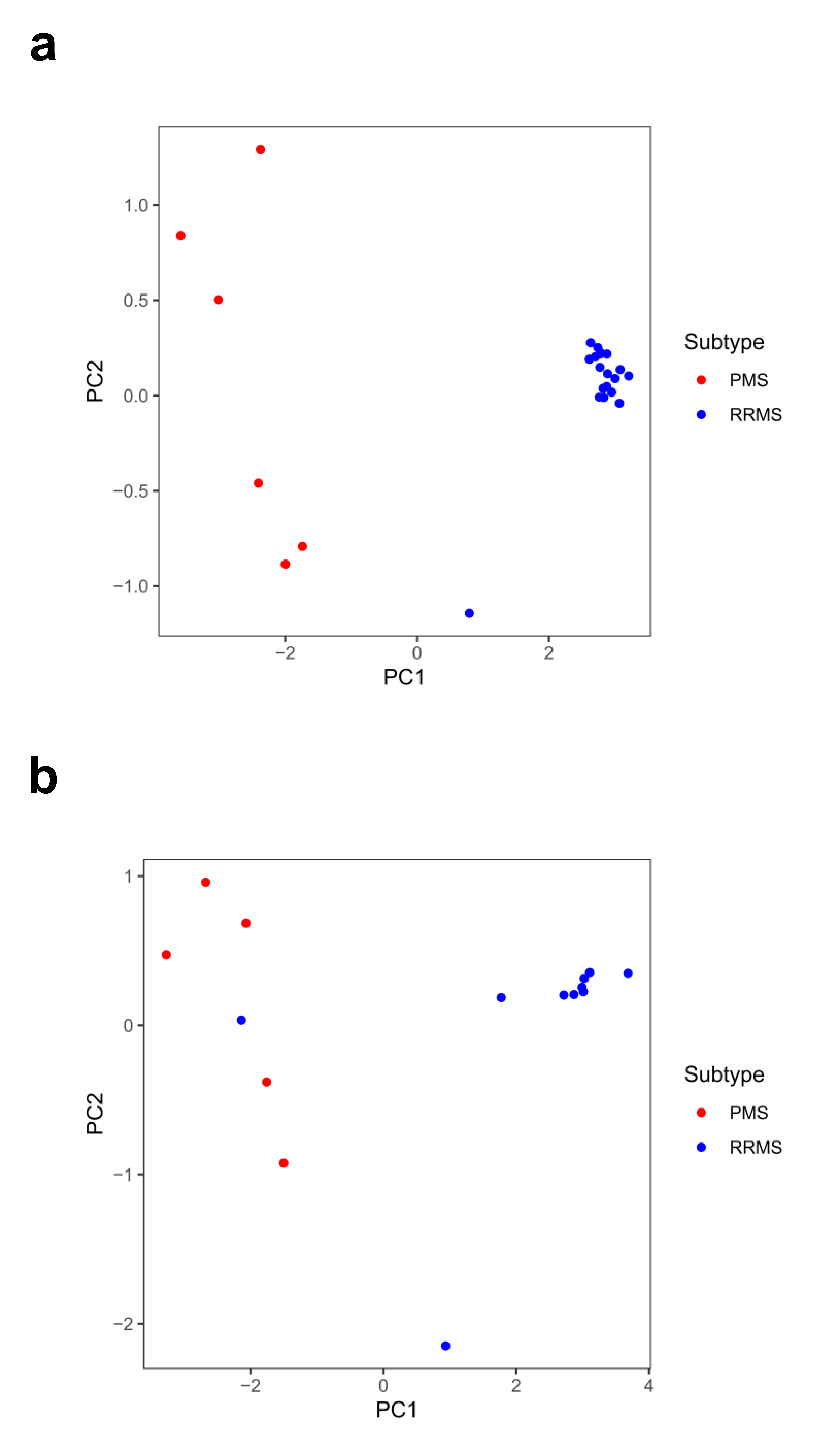

Supplement: Supplementary file 4 — Additional file 4: Figure S2. The PCA analysis was applied to evaluation of the performance of SVM classifiers based on the correlation-based distances in the training set and in the test set. [file 12967_2021_2981_MOESM4_ESM.tif]
